# Surfactin Inhibits Osteoclast Differentiation by Negatively Regulating the Elk1-AP-1-NFATc1 Axis

**DOI:** 10.3390/biomedicines14010155

**Published:** 2026-01-11

**Authors:** Kazuki Maruyama, Ayaka Koga, Yuki Kodama, Ryota Yamasaki, Yoshie Nagai-Yoshioka, Jun J. Miyamoto, Kayoko Kuroishi, Kaori Gunjigake, Tatsuo Kawamoto, Wataru Ariyoshi

**Affiliations:** 1 Division of Orofacial Functions and Orthodontics, Department of Craniofacial Growth and Development, Kyushu Dental University, Kitakyushu 803-8580, Fukuoka, Japan; r21maruyama@fa.kyu-dent.ac.jp (K.M.); r24miyamoto@fa.kyu-dent.ac.jp (J.J.M.); kayo-na@kyu-dent.ac.jp (K.K.); k-kaori@kyu-dent.ac.jp (K.G.); r15kawamoto@fa.kyu-dent.ac.jp (T.K.); 2 Division of Infections and Molecular Biology, Department of Advanced Pathophysiological Science, Kyushu Dental University, Kitakyushu 803-8580, Fukuoka, Japan; r20koga@fa.kyu-dent.ac.jp (A.K.); r21kodama@fa.kyu-dent.ac.jp (Y.K.); r18yamasaki@fa.kyu-dent.ac.jp (R.Y.); r16yoshioka@fa.kyu-dent.ac.jp (Y.N.-Y.); 3 Department of Health Sciences, Kyushu Dental University, Kitakyushu 803-8580, Fukuoka, Japan; 4 Oral Medicine Innovation Center, Kyushu Dental University, Kitakyushu 803-8580, Fukuoka, Japan

**Keywords:** osteoclast, surfactin, Elk1, AP-1, NFATc1

## Abstract

**Background/Objectives**: Surfactin is a biosurfactant with various biological activities, including antibacterial and anti-inflammatory properties; however, its effects on bone metabolism remain poorly understood. This study aimed to investigate the effects of surfactin on osteoclast differentiation and elucidate its underlying molecular mechanisms. **Methods**: RAW264.7 cells were treated with receptor activator of nuclear factor-kappa B ligand (RANKL) and surfactin, and osteoclast differentiation and maturation were evaluated by tartrate-resistant acid phosphatase and F-actin staining, respectively. Gene expression of differentiation markers was assessed using real-time reverse transcription-quantitative polymerase chain reaction, while the kinetics of intracellular signaling molecules and transcription factors were analyzed using Western blot analysis. **Results**: Surfactin treatment significantly inhibited osteoclast differentiation and maturation, as well as the mRNA expression of *Nfatc1*, *Acp5*, and *Cathepsin K*. Although surfactin did not markedly affect RANKL-induced activation of the NF-κB or MAPK-mediated signaling, it significantly suppressed the expression of c-Fos at both the mRNA and protein levels. Furthermore, surfactin attenuated the phosphorylation of Elk1, a transcription factor involved in c-Fos induction. **Conclusions**: Surfactin inhibits RANKL-induced osteoclast differentiation by negatively regulating the Elk1-AP-1-NFATc1 axis. Surfactin may thus be a promising therapeutic candidate for the treatment of metabolic bone disorders and inflammatory bone destruction.

## 1. Introduction

Osteoclasts are multi-nucleated giant cells that play a crucial role in bone remodeling by mediating bone resorption and maintaining bone homeostasis [1]. The differentiation of osteoclasts requires the binding of receptor activator of nuclear factor-kappa B (RANK), a member of the tumor necrosis factor receptor (TNFR) superfamily, to RANK-ligand (RANKL), which is expressed on the surface of osteoblasts and mesenchymal cells. Direct binding of RANKL to RANK, which is expressed on the surface of osteoclast precursor cells derived from the monocyte-macrophage lineage, activates intracellular signaling molecules such as nuclear factor-kappa B (NF-κB), mitogen-activated protein kinase (MAPK), and activator protein-1 (AP-1), thereby inducing osteoclast differentiation [2].

A disruption of this balance in bone remodeling leads to metabolic bone diseases such as osteoporosis and Paget’s disease, as well as inflammatory bone destruction seen in conditions such as rheumatoid arthritis and periodontal disease. Recently, as the intricate regulatory mechanisms of bone remodeling controlled by various hormones and local factors have been elucidated, preventive and therapeutic approaches aimed at maintaining bone metabolic homeostasis are being developed [3]. For metabolic bone diseases and bone metastases from cancer, drugs that inhibit osteoclast function or differentiation, such as bisphosphonates and anti-RANKL antibodies, are widely used. While these drugs ameliorate the disease conditions, they reportedly cause serious side effects such as medication-related osteonecrosis of the jaw and osteomyelitis of the jaw, leaving safety concerns unresolved [4,5]. In cases of inflammatory bone destruction, biological agents targeting inflammatory cytokines are used, but challenges remain regarding adverse effects and high cost. Therefore, there is a crucial need for new treatment approaches for bone diseases with high biosafety.

Biosurfactants are natural surfactants found in plants and animals, as well as surfactant substances produced by microbial metabolism [6]. Biosurfactants are primarily classified into glycolipid, lipopeptide/lipoproteins, phospholipid/fatty acids/neutral lipids, and polymeric biosurfactants [7]. Biosurfactants possess characteristics such as low toxicity, high biodegradability, and environmental compatibility compared to chemically synthesized products, leading to their increasing application in food, cosmetics, pharmaceuticals, and other fields.

Surfactin is a lipopeptide-type biosurfactant produced by *Bacillus* bacteria, consisting of a cyclic peptide composed of seven amino acids and a hydrophobic hydrocarbon chain [8,9]. It reportedly exhibits antimicrobial activity against *Clostridium perfringens* [10] and *Candida albicans* [11], with pore formation contributing to the destabilization of membrane structures [12]. Additionally, various biological activities, including anticancer effects and suppression of chronic inflammatory responses, have been identified [13,14]. Recently, research by Kuang et al. revealed that surfactin inhibits osteoclast differentiation by suppressing the NF-κB signaling pathway [15]. However, the effects of surfactin on bone metabolism remain poorly understood. Therefore, this study aimed to investigate the effects of surfactin on osteoclast differentiation and to elucidate the detailed molecular mechanisms involved.

## 2. Materials and Methods

### 2.1. Reagents and Antibodies

Surfactin sodium salt with 96.7% purity from *Bacillus subtilis* was supplied by Kaneka Corporation (Tokyo, Japan) and dissolved in α-modified minimum essential medium (Fujifilm Wako Pure Chemical Corporation, Osaka, Japan) containing 100 units/mL penicillin, 100 μg/mL streptomycin (Fujifilm Wako Pure Chemical Corporation), and 10% fetal bovine serum (Sigma-Aldrich, St. Louis, MO, USA). Recombinant human soluble RANKL was obtained from PeproTech (Cranbury, NJ, USA) and Oriental Yeast Co., Ltd. (Tokyo, Japan). Anti-NF-κB p65 (D14E12), anti-IκBα (L35A5), anti-phospho-p38 MAPK, anti-p38 MAPK, anti-phospho-ERK1/2 (D13.14.4E), anti-ERK1/2 (137F5), anti-phospho-JNK, anti-JNK, anti-phospho-c-jun (54B3), anti-c-jun (60A8), anti-phospho-c-Fos (D82C12), anti-c-Fos (9F6), and anti-Histone H3 antibodies were purchased from Cell Signaling Technology Inc. (Beverly, MA, USA). Anti-β-actin (AC-15) antibody was purchased from Sigma-Aldrich. Anti-Elk1 (ab218133) and anti-Elk1 (E277) antibodies were purchased from Abcam (Cambridge, UK).

### 2.2. Cell Culture

RAW264.7 cells (RIKEN BRC Cell Bank: RCB0535, Tsukuba, Japan), monocyte macrophages derived from mice, were used as osteoclast progenitor cells. RAW264.7 cells were cultured using previously reported methods [16].

### 2.3. Cell Counting Kit-8 (CCK-8) Assay

Cells were seeded at 4 × 10^4^ cells/well in a 96-well culture plate. After 24 h, cells were stimulated with different concentrations of surfactin for 48 h. Subsequently, the cell counting kit-8 (CCK-8) assay was performed to evaluate cell viability. CCK-8 solution (Dojindo Molecular Technologies Inc., Kumamoto, Japan) was added to each well, and the cells were incubated at 37 °C for 1 h. The absorbance of each well was measured at a wavelength of 450 nm using a microplate spectrophotometer (MultiskanFC; ThermoFisher Scientific Inc., Waltham, MA, USA).

### 2.4. TRAP Staining

Cells were seeded at 3.5 × 10^3^ cells/well in 96-well plates. After 24 h, seeded cells were stimulated with 100 ng/mL RANKL and 25 μg/mL surfactin for 6 days. The medium was replaced every 48 h. Following the 6 days after inducing osteoclast differentiation, the cells were fixed, washed three times with phosphate-buffered saline, and then incubated in tartrate-resistant acid phosphatase (TRAP) staining solution at 37 °C for 1.5 h in the dark according to the instructions of the TRAP staining kit (Sigma-Aldrich). TRAP-positive multi-nucleated cells with ≥3 nuclei were counted as osteoclasts under light microscopy (IX71; Olympus Corporation, Tokyo, Japan). Additionally, the area of TRAP-positive cells was quantified using a fluorescent and phase contrast microscope (BZ-X810; KEYENCE, Osaka, Japan).

### 2.5. Animal Experiments

Osteoclast precursor cells were prepared from bone marrow cells of eight-week-old male ddY mice (Japan SLC, Shizuoka, Japan) using previously reported methods [17]. Osteoclast precursor cells were seeded at a density of 2.5 × 10^5^ cells/mL in 48-well plates and cultured with recombinant human macrophage colony-stimulating factor (rhM-CSF; 20 ng/mL; PeproTech), RANKL (100 ng/mL), and surfactin (25 μg/mL) at 37 °C in a 5% CO_2_ atmosphere for 72 h. TRAP staining was performed as described above and the stained cells were observed. The experiments performed in this study were approved by the Kyushu Dental University Experimental Animal Care and Use Committee (approval number: 25-028).

### 2.6. F-Actin Staining

Cells were seeded at 1.86 × 10^4^ cells/well in a chamber slide (WATSON, Tokyo, Japan). After 24 h, cells were stimulated with 100 ng/mL RANKL and 25 μg/mL surfactin for 6 days. The medium was replaced every 48 h. Following the 6-day induction of osteoclastogenesis, the cells were fixed using 4% paraformaldehyde for 10 min. For F-actin ring visualization, cells were permeabilized with 0.2% Triton X-100 (Merck Millipore, MA, USA) for 5 min, blocked with 1% bovine serum albumin, and incubated with Alexa Fluor 568 phalloidin (Thermo Fisher Scientific Inc.) for 20 min in darkness to label filamentous actin. Nuclei were counterstained with Antifade Mounting Medium with 4′,6-diamidino-2-phenylindole (DAPI) (Vector Laboratories, Newark, CA, USA) for 5 min. Fluorescent images were acquired using a fluorescent and phase contrast microscope (BZ-X810, excitation/emission: 578 nm/600 nm for phalloidin; 360 nm/460 nm for DAPI), and the area of F-actin-stained multinucleated cells containing at least three nuclei was quantified.

### 2.7. Real-Time RT-qPCR

Total RNA was isolated using the Cica Geneus RNA Prep Kit (Kanto Chemical Co., Inc., Tokyo, Japan), according to the manufacturer’s instructions. Real-time reverse transcription-polymerase chain reaction (RT-qPCR) was used to quantify mRNA expression. Reverse transcription for cDNA and real-time RT-qPCR were carried out as previously described [18]. Specific primers used for real-time RT-qPCR are shown in Table 1.

### 2.8. Western Blot Analysis

Whole cell lysates were prepared using Cell Lysis Buffer (Cell Signaling Technology Inc.) supplemented with protease inhibitors (Thermo Fisher Scientific Inc.). For the detection of NF-κB p65, proteins from the nuclear and cytoplasmic fractions were extracted separately using NE-PER Nuclear and Cytoplasmic Extraction Reagent (Thermo Fisher Scientific Inc.), according to the manufacturer’s protocol. Protein concentration measurement, electrophoresis, and transfer to membrane were performed according to previously described methods [16]. Anti-phospho-p38 MAPK, anti-p38 MAPK, anti-phospho-ERK1/2, anti-ERK1/2, anti-phospho-JNK, anti-JNK, anti-phospho-c-jun, anti-c-jun, anti-phospho-c-Fos, anti-c-Fos, Histone H3, anti-Elk1, anti-Elk1 antibody, and anti-β-actin antibodies were used as primary antibodies. Rabbit IgG horse-radish peroxidase (HRP)-linked whole Ab (from donkey) and mouse IgG HRP-linked whole Ab (from sheep) were used as secondary antibodies (Cytiva, Tokyo, Japan). Signal detection was performed by generating chemiluminescence using an enhanced chemiluminescence reagent (Amersham Biosciences, Uppsala, Sweden) and captured by a digital system (GelDoc XR Plus; Bio-Rad, CA, USA) equipped with Image LabTM^®^ 2.0 software (Bio-Rad). The band intensity of each blot was quantified by densitometric analysis using Image LabTM^®^ 2.0 software (Bio-Rad).

### 2.9. Statistical Analyses

The comparison of the mean values of each group was performed using analysis of variance. The statistical significance level was set at *p* < 0.05, and Dunnett’s or Tukey’s test was used to test for significant differences. For comparison between two groups, Student’s two-tailed t-test was used. EZR software version 1.54 was used for statistical analyses [19].

## 3. Results

### 3.1. Surfactin Inhibits Cell Proliferation at High Concentrations

**Figure 1:**
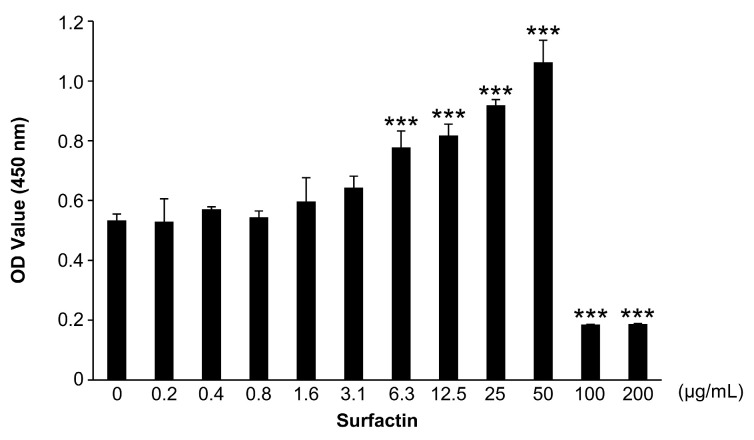
Effects of surfactin on the proliferation of RAW264.7 cells. RAW264.7 cells were stimulated with the indicated concentrations of surfactin (0–200 μg/mL) for 48 h. Viable cells were detected by the cell counting kit-8 (CCK-8) assay (*n* = 3). Data were analyzed using Dunnett’s test following one-way ANOVA. *** *p* < 0.001.

The effect of surfactin on RAW264.7 cell proliferation was evaluated using the CCK-8 assay (Figure 1). Within the range of 50–100 μg/mL, no effect of surfactin on cell proliferation was observed, whereas in groups treated with concentrations above 100 μg/mL, the number of viable cells significantly reduced.

### 3.2. Surfactin Inhibits Osteoclast Formation

**Figure 2:**
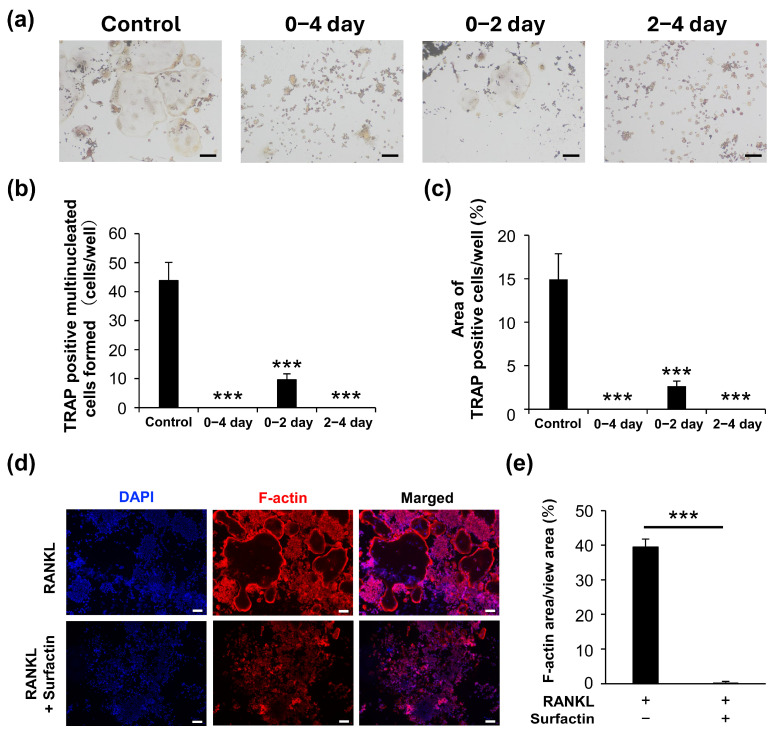
Surfactin inhibits osteoclast differentiation induced by RANKL in RAW264.7. (**a**) RAW264.7 cells were stimulated with or without surfactin (25 μg/mL) in the presence of RANKL (100 ng/mL) for 4 days and then subjected to tartrate-resistant acid phosphatase (TRAP) staining. Scale bar = 100 μm. (**b**) TRAP-positive multi-nucleated cells (containing more than three nuclei) were counted (*n* = 3). (**c**) The area of TRAP-positive cells was quantified (*n* = 3). (**d**) RAW264.7 cells were stimulated with or without surfactin (25 μg/mL) in the presence of RANKL (100 ng/mL) for 5 days and then subjected to F-actin staining. Scale bar = 100 μm. (**e**) The area of F-actin-stained multinucleated cells was quantified (*n* = 3). Multiple data were analyzed using the Dunnett’s test after one-way ANOVA (**b**,**c**). For two-group comparisons, significance was tested using a two-tailed *t*-test. *** *p* < 0.001.

**Figure 3:**
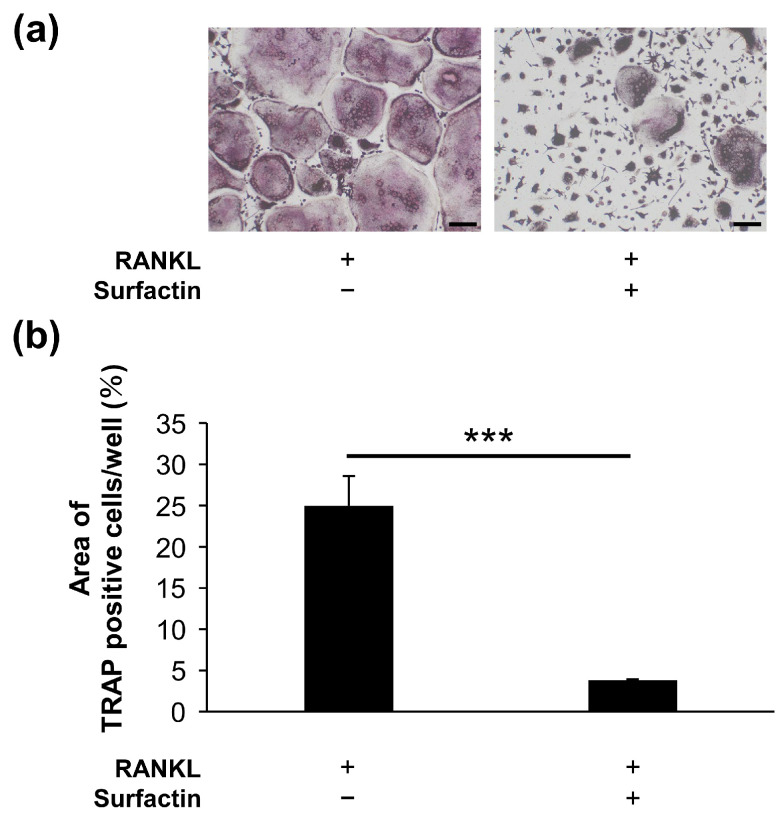
Surfactin inhibits osteoclast differentiation induced by RANKL in bone marrow-derived osteoclast precursor cells. (**a**) Osteoclast precursor cells were stimulated with or without Surfactin (25 μg/mL) in the presence of RANKL (100 ng/mL) for 2 days and then subjected to tartrate-resistant acid phosphatase (TRAP) staining. Scale bar = 100 μm. (**b**) The area of TRAP-positive cells was quantified (*n* = 3). Significance was tested using a two-tailed *t*-test. *** *p* < 0.001.

The effect of surfactin on osteoclast formation in RAW264.7 cells was evaluated using TRAP staining. Surfactin administration significantly reduced the number of TRAP-positive multi-nucleated cells induced by RANKL. We also examined the differentiation process of RAW264.7 cells during 4-day RANKL stimulation in the presence or absence of surfactin. We observed that stimulation of surfactin inhibited RANKL-induced osteoclast differentiation during both the early stage (days 0–2) and later stages (days 2–4) of differentiation (Figure 2a–c). To assess the effect of surfactin on osteoclast maturation, F-actin staining was performed. Surfactin inhibited the formation of F-actin rings induced by RANKL (Figure 2d,e). In addition to RAW264.7 cells, a notable reduction in the area TRAP-positive multinucleated cells differentiated from bone marrow cells was observed by the addition of 25 μg/mL surfactin, compared to the RANKL-stimulated control cells (Figure 3).

### 3.3. Surfactin Reduces the Expression of RANKL-Induced Osteoclast Differentiation Marker Genes

**Figure 4:**
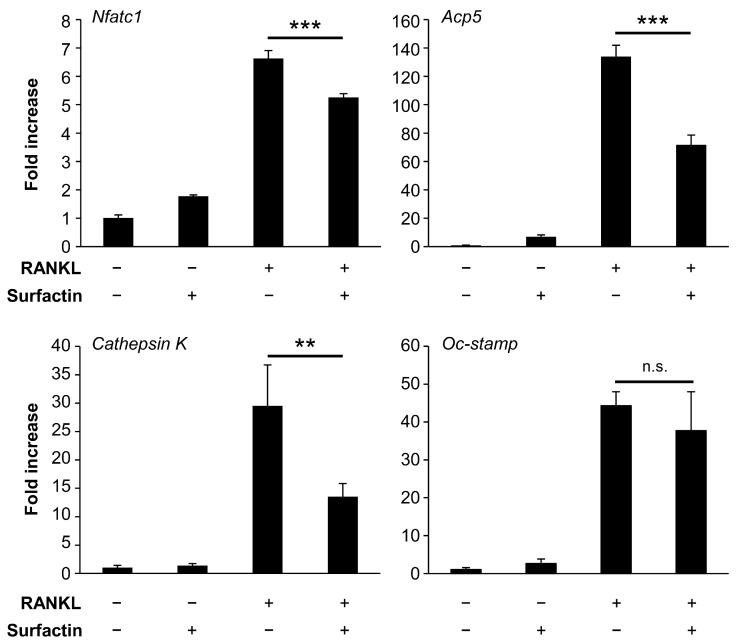
Effect of surfactin on gene expression in RAW264.7 cells. RAW264.7 cells were stimulated with surfactin (25 μg/mL) in the presence or absence of RANKL (100 ng/mL) for 48 h. The expression levels of *Nfatc1*, *Acp5*, *Cathepsin K*, and *Oc-stamp* were quantified using real-time reverse transcription quantitative polymerase chain reaction (*n* = 3). Data were analyzed using Tukey’s test following one-way ANOVA. ** *p* < 0.01 and *** *p* < 0.001. n.s., no significance.

To evaluate the effect of surfactin treatment on gene expression during osteoclast differentiation, we examined the mRNA levels of osteoclast differentiation markers (*Nfatc1*, *Acp5*, *Cathepsin K*, and *Oc-stamp*) (Figure 4). The mRNA levels of *Nfatc1*, *Acp5*, and *Cathepsin K* were significantly reduced by surfactin treatment compared to RAW264.7 cells treated with RANKL. In contrast, no significant change in RANKL-induced *Oc-stamp* mRNA levels was observed following surfactin treatment.

### 3.4. Surfactin Has No Effect on Activation of the NF-κB Pathway Induced by RANKL

**Figure 5:**
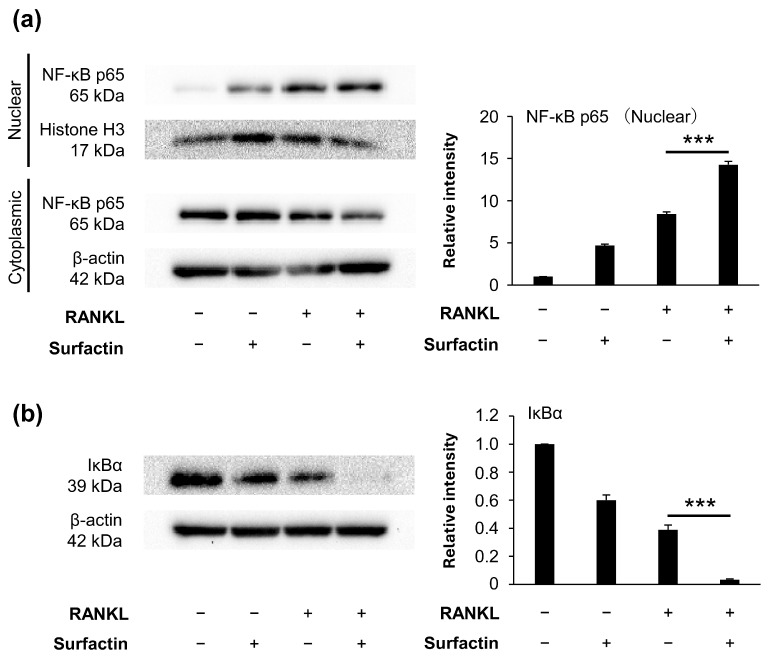
Effects of surfactin on activation of the NF-κB pathway in RAW264.7 cells. RAW264.7 cells were incubated with or without surfactin (25 μg/mL) in the presence or absence of RANKL (100 ng/mL) for 15 min. (**a**) Protein expression of NF-κB p65 in nuclear and cytoplasmic fractions was detected by Western blot analysis. Histone H3 (nuclear fraction) and β-actin (cytoplasmic fraction) were used as loading controls. Relative band intensities of p65 protein in nuclear fraction normalized to changes in the Histone H3 protein were quantified by densitometric analyses (*n* = 3). (**b**) Protein expression of IκBα in whole cell lysates was also detected by Western blot analysis. β-actin was used as a loading control. Relative band intensities of IκBα protein normalized to changes in the β-actin protein were quantified by densitometric analyses (*n* = 3). Data were analyzed using Tukey’s test following one-way analysis of variance (ANOVA). *** *p* < 0.001.

To elucidate the molecular mechanism by which surfactin inhibits osteoclast formation, we focused on the activation of the RANKL-induced NF-κB pathway. We have found that protein degradation of IκBα is induced 15 min after RANKL stimulation (Supplemental Figure S1). However, no inhibitory effect of surfactin administration was observed on either the RANKL-induced nuclear translocation of NF-κB p65 (Figure 5a) or the degradation of IκBα protein (Figure 5b).

### 3.5. Surfactin Has No Effect on the Activation of the MAPK Pathway Induced by RANKL

**Figure 6:**
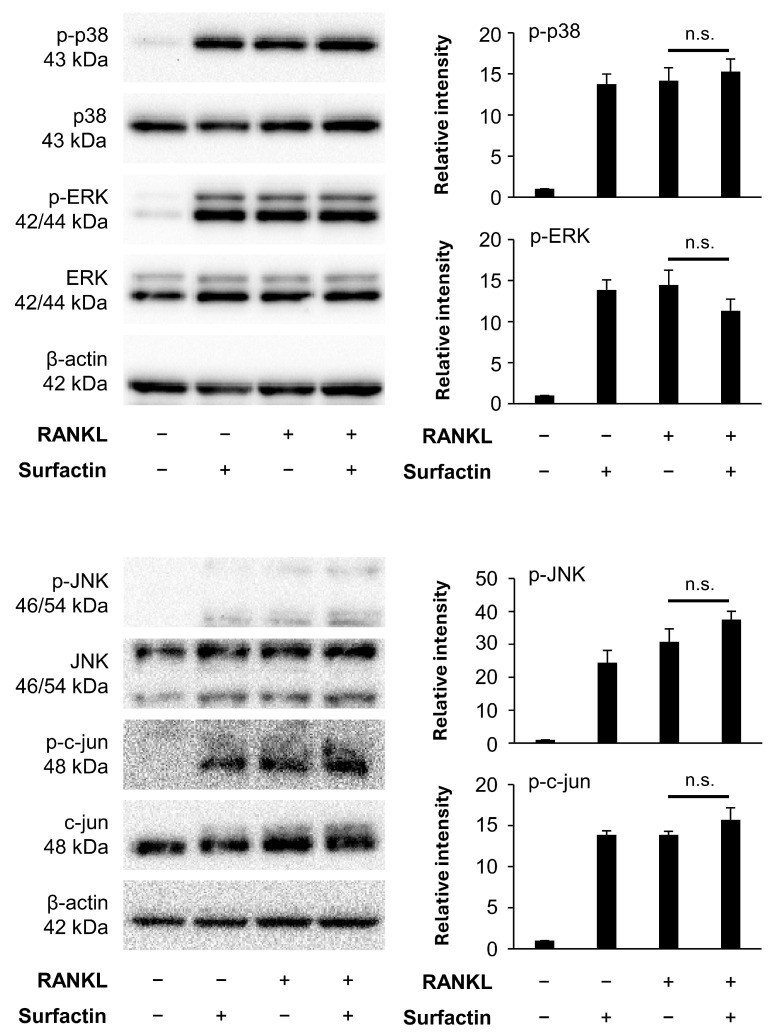
Effects of surfactin on the MAPK-mediated signaling pathway activated by RANKL. RAW264.7 cells were stimulated with RANKL (100 ng/mL) and surfactin (25 μg/mL) for 15 min. Protein expression of phosphorylated p38 MAPK (p-p38), p38 MAPK (p38), phosphorylated ERK (p-ERK), ERK, phosphorylated JNK (p-JNK), JNK, phosphorylated c-jun (p-c-jun), and c-jun was detected by Western blot analysis. β-actin was used as a loading control. Relative band intensities of each phosphorylated protein normalized to changes in the total protein were quantified by densitometric analyses (*n* = 3). Data were analyzed using Tukey’s test following one-way analysis of variance (ANOVA). n.s., no significance.

Next, we focused on the activation of the RANKL-induced MAPK pathway. RANKL stimulation also transiently enhanced the phosphorylation of molecules constituting the MAPK pathway (p38, ERK, and JNK) in RAW264.7 cells, reaching a peak within 15 min (Supplemental Figure S2). Surfactin did not affect RANKL-induced phosphorylation of p38, ERK, JNK, or c-jun (Figure 6).

### 3.6. Surfactin Inhibits Activation of the Elk1-c-Fos Axis Induced by RANKL

**Figure 7:**
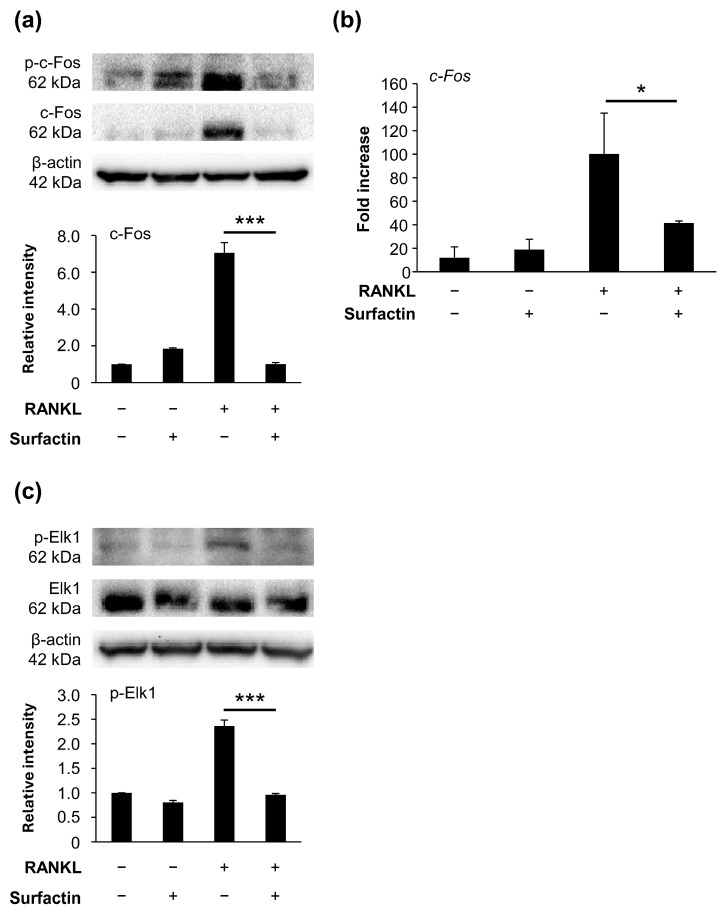
Effects of surfactin on RANKL-induced expression and activation of Elk1/c-Fos axis. (**a**) RAW264.7 cells were stimulated with RANKL (100 ng/mL) and surfactin (25 μg/mL) for 48 h. Protein expression of phosphorylated c-Fos (p-c-Fos) and c-Fos was detected by Western blot analysis. β-actin was used as a loading control. Relative band intensities of total c-Fos protein normalized to changes in the β-actin protein were quantified by densitometric analyses (*n* = 3). (**b**) RAW264.7 cells were stimulated with RANKL (100 ng/mL) and surfactin (25 μg/mL) for 24 h. The mRNA expression of *c-Fos* was detected by real-time reverse transcription quantitative polymerase chain reaction (RT-qPCR) analysis (*n* = 3). (**c**) RAW264.7 cells were stimulated with RANKL (100 ng/mL) and surfactin (25 μg/mL) for 1 h. The protein expression levels of phosphorylated Elk1 (p-Elk1) and Elk1 were detected by Western blot analysis. β-actin was used as a loading control. Relative band intensities of each phosphorylated Elk1 protein normalized to changes in the total Elk1 protein were quantified by densitometric analyses (*n* = 3). Data were analyzed using Tukey’s test following one-way analysis of variance (ANOVA). * *p* < 0.05 and *** *p* < 0.001.

Focusing on c-Fos, a transcription factor downstream of the MAPK pathway, we investigated the effects of surfactin on c-Fos expression. During the RANKL-induced osteoclast differentiation, surfactin suppressed both the phosphorylation and expression of c-Fos protein (Figure 7a). The mRNA level of *c-Fos* was also significantly reduced in the surfactin-treated group compared to the group treated with RANKL alone (Figure 7b). Therefore, we focused on the activation of Elk1, a transcription factor functioning downstream of MAPK that regulates c-Fos expression. We found that surfactin inhibited the phosphorylation of Elk1 induced by RANKL (Figure 7c).

## 4. Discussion

Surfactin is reportedly cytotoxic against cancer cells such as breast cancer, colorectal cancer, and leukemia [20]. In this study, no significant inhibition of proliferation was observed in the osteoclast precursor cell line RAW264.7 cells at surfactin concentrations ranging from 0 to 50 μg/mL (Figure 1). Based on this result, we increased subsequent surfactin concentrations by 25 μg/mL to examine its ability to modify osteoclast differentiation.

Administration of surfactin to RAW264.7 cells and bone marrow cells significantly suppressed osteoclast differentiation and maturation induced by RANKL (Figure 2 and Figure 3). Interestingly, the number of osteoclasts formed significantly reduced even in groups treated with surfactin only during the early phase (0–2 days) or only during the late phase (2–4 days) of the differentiation process. These results suggest that surfactin negatively regulates multiple stages of osteoclast differentiation. This study focused specifically on the inhibitory effect of surfactin during the early stages of osteoclast differentiation, aiming to elucidate its detailed molecular mechanism.

The binding of RANKL to RANK on the precursor cell membrane enhances the expression of NFATc1, the master regulator of osteoclast differentiation, through the activation of various intracellular signaling pathways [21]. Activated NFATc1 synergizes with other transcription factors and plays an essential role in inducing the expression of genes crucial for osteoclast function, such as ACP5 and Cathepsin K [22,23]. The addition of surfactin significantly reduced the mRNA expression of *Nfatc1*, *Acp5*, and *Cathepsin K* induced by RANKL (Figure 4), suggesting that the negative regulation of osteoclast differentiation by surfactin involves suppression of NFATc1 expression induced by RANK-RANKL interaction.

The induction of NFATc1 expression involves the activation of transcription factors such as NF-κB and AP-1. In the NF-κB pathway, RANKL stimulation induces the phosphorylation and degradation of the IκBα protein, resulting in the release of the p65/p50 heterodimer, which translocates into the nucleus and exerts transcriptional activity [24]. In contrast, AP-1 transcriptional activity in RANKL-induced osteoclast differentiation is induced by activating the MAPK pathway, by inducing ERK, p38, and JNK [25]. However, the activation of the NF-κB and MAPK pathways induced by RANKL was not suppressed by surfactin administration. Furthermore, the phosphorylation level of c-jun protein, a downstream molecule of JNK, was also not modified by surfactin (Figure 5 and Figure 6). Based on these results, we hypothesized that the target molecule involved in the suppression of negative regulation of osteoclast differentiation by surfactin is a downstream molecule of MAPK that regulates AP-1 transcription activity. Surprisingly, administration of surfactin alone slightly activated NF-κB and MAPK signaling pathways in RAW264.7 cells, but did not affect NFATc1 expression or osteoclast differentiation (Figure 5, Figure 6 and Figure 7). Surfactin derived from *Bacillus amyloliquefaciens* has been reported to modulate the innate immune response in RAW264.7 cells by activating various signaling pathways, including NF-κB and MAPK, and to promote inflammasome activation [26]. The biological effects of surfactin-mediated activation of these signaling pathways on osteoclast precursor cells require further investigation.

c-Fos, a transcription factor essential for osteoclast differentiation, forms the heterodimer AP-1 with c-jun [21,27] and binds to the promoter region of NFATc1 [28]. Co-stimulation with surfactin negatively regulated c-Fos protein and mRNA expression induced in RANKL-stimulated osteoclast precursor cells (Figure 7a,b). These results suggest that surfactin-mediated suppression of *c-Fos* transcription is not associated with detectable MAPK modulation at the examined time points.

Elk1 is a transcription factor belonging to the ternary complex factor subfamily. MAPK phosphorylates Elk1, forms a complex with serum response factor, and induces c-Fos expression by binding to the serum response element [29,30]. The addition of surfactin suppressed the phosphorylation of Elk1 protein in RANKL-stimulated osteoclast precursor cells (Figure 7c). These results suggest that surfactin may negatively regulate NFATc1 expression via c-Fos by directly inhibiting Elk1 activation. cAMP response element-binding protein (CREB) is reportedly also involved in the regulation of c-Fos expression in addition to Elk1 [31,32]. However, administration of surfactin to osteoclast precursor cells did not alter the level of CREB phosphorylation induced by RANKL (Supplemental Figure S3). To elucidate the detailed molecular mechanisms by which surfactin inhibits osteoclast differentiation, future studies must give attention to its direct interaction with Elk1 proteins.

Kuang et al. also demonstrated that surfactin possesses the bioactivity to inhibit RANKL-induced osteoclast differentiation in RAW264.7 cells and bone marrow cells [15]. However, unlike this study, they suggested the involvement of a negative regulatory mechanism targeting the NF-κB pathway as the system by which surfactin inhibits osteoclast differentiation. It is difficult to fully explain this discrepancy, but we speculate that it may be due to differences in the composition of surfactin based on the purification process. Due to differences in amino acid sequences and the hydrocarbon chain, there are multiple analogs of surfactin [33], and this diversity affects parameters such as surfactant activity, hemolytic activity [34,35], antitumor activity [20,36], and antimicrobial activity [37]. In this study, we used mixtures of six surfactin analogs, each composed of a hydrophobic moiety with three different alkyl chain lengths and two different terminal branching patterns (iso and anteiso). Previously a structural analysis by RP-HPLC and MALDI TOF mass spectrometry revealed that surfactin using in this study has a fatty acid composition of C13 (17%), C14 (52%) and C15 (31%) [38,39]. From the perspective of the composition of analogs, future studies focusing on the molecular mechanisms of the negative regulation of osteoclast differentiation by surfactin are required.

In this study, administration of surfactin during the late stage of osteoclast differentiation (days 2–4) significantly suppressed osteoclast formation (Figure 2), suggesting that surfactin may inhibit not only intracellular signaling induced at the early differentiation phase but also the cell–cell fusion process occurring during late differentiation. However, the gene expression of *Oc-stamp*, a multi-pass transmembrane protein involved in the fusion of precursor cells during osteoclast differentiation induction [40,41,42,43], showed no significant suppression following surfactin administration (Figure 4). In addition to OC-STAMP, several factors such as DC-STAMP [44], v-ATPase V0 subunit d2 [45], and CD47 [46] have been reported to be involved in cell fusion during the osteoclast differentiation process. Furthermore, it has been demonstrated that the biosynthesis and rearrangement of phospholipids, the principal components of the cell membrane, are also essential for the fusion of osteoclasts [47,48]. Research focusing on the various osteoclast fusiona-related factors and on the dynamics of phospholipids in cell membranes has led to the elucidation of the negative regulatory mechanism in the cell fusion process of surfactin.

A limitation of this study is that the effect of surfactin on the RANKL-activated signaling pathway involved in the osteoclast formation was evaluated at only one time point. Analyses of intracellular signal modification over time are also necessary in the future. Furthermore, the results of present studies are confined to analyses using a single cell lineage (RAW264.7 cells). In addition to molecular biological analyses using primary cell cultures, verification of the effect of surfactin on bone loss in animal models, including osteoporosis and periodontitis, is required for clinical application.

## 5. Conclusions

**Figure 8:**
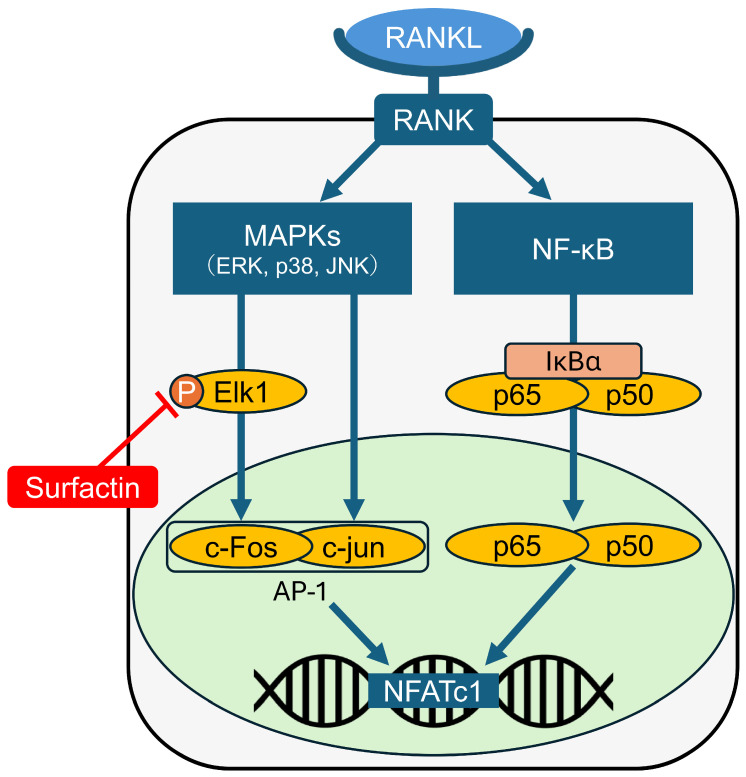
Schematic representation of the molecular mechanisms down-regulating NFATc1 expression induced by surfactin in osteoclast progenitors.

We demonstrated that surfactin suppresses RANKL-induced osteoclast differentiation by negatively regulating the Elk1-AP-1-NFATc1 axis (illustrated in Figure 8). This suggests that surfactin may be a promising candidate for application in novel therapeutic approaches targeting metabolic bone diseases and inflammatory bone destruction.

## Figures and Tables

**Table 1:** Real-time RT-qPCR primer sequences.

Gene		Primer Sequence (5′-3′)
*Gapdh*	forward reverse	5′- GAC GGC CGC ATC TTC TTT GA -3′ 5′- CAC ACC GAC CTT CAC CAT TTT -3′
*Nfatc1*	forward reverse	5′-ACC ACC TTT CCG CAA CCA-3′ 5′-GGT ACT GGC TTC TCT TCC GTT TC-3′
*Acp5*	forward reverse	5′-GCA GTA TCT TCA GGA CGA GAA C-3′ 5′-TCC ATA GTG AAA CCG CAA GTA G-3′
*Oc-stamp*	forward reverse	5′-CCG CAG CCT GAC ATT TGA G-3′ 5′- TCT CCT GAG TGA TCG TGT GCA T -3′
*Cathepsin K*	forward reverse	5′-TAT GAC CAC TGC CTT CCA ATA C-3′ 5′-GCC GTG GCG TTA TAC ATA CA-3′
*c-Fos*	forward reverse	5′-GCC AAG TGC CGG AAT CG-3′ 5′-CAA CGC AGA CTT CTC ATC TTC AA-3′

## Data Availability

The raw data supporting the conclusions of this article will be made available by the corresponding author on request.

## Supplementary Materials

The following supporting information can be downloaded at: https://www.mdpi.com/article/10.3390/biomedicines14010155/s1, Figure S1: Time-dependent effects of RANKL on NF-κB activation.; Figure S2: Time-dependent effects of RANKL on MAPK activation.; Figure S3: Effects of surfactin on CREB phosphorylation activated by RANKL.

## Abbreviations

The following abbreviations are used in this manuscript:

RT-qPCR	reverse transcription quantitative polymerase chain reaction
RANK	receptor activator of nuclear factor-kappa B
RANKL	receptor activator of nuclear factor-kappa B ligand
TNFR	tumor necrosis factor receptor
MAPK	mitogen-activated protein kinase
AP-1	activator protein-1
NF-κB	nuclear factor-kappa B
TRAP	tartrate-resistant acid phosphatase

## References

[1] Kim J.M., Lin C., Stavre Z., Greenblatt M.B., Shim J.H. (2020). Osteoblast-Osteoclast Communication and Bone Homeostasis. Cells.

[2] Park J.H., Lee N.K., Lee S.Y. (2017). Current Understanding of RANK Signaling in Osteoclast Differentiation and Maturation. Mol. Cells.

[3] Xu H., Wang W., Liu X., Huang W., Zhu C., Xu Y., Yang H., Bai J., Geng D. (2023). Targeting strategies for bone diseases: Signaling pathways and clinical studies. Signal Transduct. Target. Ther..

[4] Rachner T.D., Khosla S., Hofbauer L.C. (2011). Osteoporosis: Now and the future. Lancet.

[5] Song S., Guo Y., Yang Y., Fu D. (2022). Advances in pathogenesis and therapeutic strategies for osteoporosis. Pharmacol. Ther..

[6] Kitamoto D., Isoda H., Nakahara T. (2002). Functions and potential applications of glycolipid biosurfactants—from energy-saving materials to gene delivery carriers. J. Biosci. Bioeng..

[7] Lourenço M., Duarte N., Ribeiro I.A.C. (2024). Exploring biosurfactants as antimicrobial approaches. Pharmaceuticals.

[8] Zeriouh H., de Vicente A., Pérez-García A., Romero D. (2014). Surfactin triggers biofilm formation of Bacillus subtilis in melon phylloplane and contributes to the biocontrol activity. Environ. Microbiol..

[9] Mongkolthanaruk W. (2012). Classification of Bacillus beneficial substances related to plants, humans and animals. J. Microbiol. Biotechnol..

[10] Horng Y.B., Yu Y.H., Dybus A., Hsiao F.S., Cheng Y.H. (2019). Antibacterial activity of Bacillus species-derived surfactin on Brachyspira hyodysenteriae and Clostridium perfringens. AMB Express.

[11] Jakab Á., Kovács F., Balla N., Tóth Z., Ragyák Á., Sajtos Z., Csillag K., Nagy-Köteles C., Nemes D., Bácskay I. (2022). Physiological and transcriptional profiling of surfactin exerted antifungal effect against Candida albicans. Biomed. Pharmacother..

[12] Carrillo C., Teruel J.A., Aranda F.J., Ortiz A. (2003). Molecular mechanism of membrane permeabilization by the peptide antibiotic surfactin. Biochim. Biophys. Acta.

[13] Zhen C., Ge X.F., Lu Y.T., Liu W.Z. (2023). Chemical structure, properties and potential applications of surfactin, as well as advanced strategies for improving its microbial production. AIMS Microbiol..

[14] Gan P., Jin D., Zhao X., Gao Z., Wang S., Du P., Qi G. (2016). Bacillus-produced surfactin attenuates chronic inflammation in atherosclerotic lesions of ApoE^−/−^ mice. Int. Immunopharmacol..

[15] Kuang Z., Yang X., Cao Z., Li Y., Hu J., Hong X., Li B., Wu C., Qi Q., Liu X. (2023). Surfactin suppresses osteoclastogenesis via the NF-κB signaling pathway, promotes osteogenic differentiation in vitro, and inhibits oestrogen deficiency-induced bone loss in vivo. Int. Immunopharmacol..

[16] Koga A., Nagai-Yoshioka Y., Yamasaki R., Adachi Y., Fujii W., Ariyoshi W. (2025). Molecular Mechanisms of Curdlan-Induced Suppression of NFATc1 Expression in Osteoclasts. J. Cell. Biochem..

[17] Ariyoshi W., Koga A., Kodama Y., Yamasaki R., Nagai-Yoshioka Y., Usui M., Mochizuki S., Adachi Y. (2025). Inhibition of osteoclast differentiation by (1→3)-β-D-glucan from Alcaligenes faecalis (curdlan) and dectin-1 interaction via the syk proteolytic system. Carbohydr. Polym. Technol. Appl..

[18] Chaweewannakorn W., Ariyoshi W., Okinaga T., Fujita Y., Maki K., Nishihara T. (2019). Ameloblastin attenuates RANKL-mediated osteoclastogenesis by suppressing activation of nuclear factor of activated T-cell cytoplasmic 1 (NFATc1). J. Cell. Physiol..

[19] Kanda Y. (2013). Investigation of the freely available easy-to-use software ‘EZR’ for medical statistics. Bone Marrow Transpl..

[20] Wu Y.S., Ngai S.C., Goh B.H., Chan K.G., Lee L.H., Chuah L.H. (2017). Anticancer activities of Surfactin and Potential Application of Nanotechnology Assisted Surfactin Delivery. Front. Pharmacol..

[21] Takayanagi H., Kim S., Koga T., Nishina H., Isshiki M., Yoshida H., Saiura A., Isobe M., Yokochi T., Inoue J. (2002). Induction and activation of the transcription factor NFATc1 (NFAT2) integrate RANKL signaling in terminal differentiation of osteoclasts. Dev. Cell..

[22] Hayman A.R., Bune A.J., Bradley J.R., Rashbass J., Cox T.M. (2000). Osteoclastic tartrate-resistant acid phosphatase (Acp 5): Its localization to dendritic cells and diverse murine tissues. J. Histochem. Cytochem..

[23] Troen B.R. (2006). The regulation of cathepsin K gene expression. Ann. N. Y. Acad. Sci..

[24] Boyce B.F., Li J., Yao Z., Xing L. (2023). Nuclear Factor-Kappa B Regulation of Osteoclastogenesis and Osteoblastogenesis. Endocrinol. Metab..

[25] Lee K., Seo I., Choi M.H., Jeong D. (2018). Roles of Mitogen-Activated Protein Kinases in Osteoclast Biology. Int. J. Mol. Sci..

[26] Gan P., Gao Z., Zhao X., Qi G. (2016). Surfactin inducing mitochondria-dependent ROS to activate MAPKs, NF-κB and inflammasomes in macrophages for adjuvant activity. Sci. Rep..

[27] Bakiri L., Takada Y., Radolf M., Eferl R., Yaniv M., Wagner E.F., Matsuo K. (2007). Role of heterodimerization of c-Fos and Fra1 proteins in osteoclast differentiation. Bone.

[28] Matsuo K., Galson D.L., Zhao C., Peng L., Laplace C., Wang K.Z., Bachler M.A., Amano H., Aburatani H., Ishikawa H. (2004). Nuclear factor of activated T-cells (NFAT) rescues osteoclastogenesis in precursors lacking c-Fos. J. Biol. Chem..

[29] Janknecht R., Ernst W.H., Pingoud V., Nordheim A. (1993). Activation of ternary complex factor Elk-1 by MAP kinases. EMBO J..

[30] Cavigelli M., Dolfi F., Claret F.X., Karin M. (1995). Induction of c-fos expression through JNK-mediated TCF/Elk-1 phosphorylation. EMBO J..

[31] Kim J.H., Kim K., Kim I., Seong S., Lee K.B., Kim N. (2018). BCAP promotes osteoclast differentiation through regulation of the p38-dependent CREB signaling pathway. Bone.

[32] Lu D.Z., Dong W., Feng X.J., Chen H., Liu J.J., Wang H., Zang L.Y., Qi M.C. (2020). CaMKII(δ) regulates osteoclastogenesis through ERK, JNK, and p38 MAPKs and CREB signalling pathway. Mol. Cell. Endocrinol..

[33] Peypoux F., Bonmatin J.M., Wallach J. (1999). Recent trends in the biochemistry of surfactin. Appl. Microbiol. Biotechnol..

[34] Dufour S., Deleu M., Nott K., Wathelet B., Thonart P., Paquot M. (2005). Hemolytic activity of new linear surfactin analogs in relation to their physico-chemical properties. Biochim. Biophys. Acta.

[35] Su Y., Gao L., Li C., Wang L., Zhou H., Zhang C., Xia X. (2024). Regulation mechanism and bioactivity characteristic of surfactin homologues with C14 and C15 fatty acid chains. Microb. Cell. Fact..

[36] Miceli R.T., Totsingan F., Naina T., Islam S., Dordick J.S., Corr D.T., Gross R.A. (2023). Molecularly Engineered Surfactin Analogues Induce Nonapoptotic-Like Cell Death and Increased Selectivity in Multiple Breast Cancer Cell Types. ACS Omega.

[37] Deleu M., Lorent J., Lins L., Brasseur R., Braun N., El Kirat K., Nylander T., Dufrêne Y.F., Mingeot-Leclercq M.P. (2013). Effects of surfactin on membrane models displaying lipid phase separation. Biochim. Biophys. Acta.

[38] Imura T., Ikeda S., Aburai K., Taira T., Kitamoto D. (2013). Interdigitated lamella and bicontinuous cubic phases formation from natural cyclic surfactin and its linear derivative. J. Oleo Sci..

[39] Taira T., Yanagisawa S., Nagano T., Zhu Y., Kuroiwa T., Koumura N., Kitamoto D., Imura T. (2015). Selective encapsulation of cesium ions using the cyclic peptide moiety of surfactin: Highly efficient removal based on an aqueous giant micellar system. Colloids Surf. B Biointerfaces.

[40] Ishii T., Ruiz-Torruella M., Ikeda A., Shindo S., Movila A., Mawardi H., Albassam A., Kayal R.A., Al-Dharrab A.A., Egashira K. (2018). OC-STAMP promotes osteoclast fusion for pathogenic bone resorption in periodontitis via up-regulation of permissive fusogen CD9. FASEB J..

[41] Witwicka H., Hwang S.Y., Reyes-Gutierrez P., Jia H., Odgren P.E., Donahue L.R., Birnbaum M.J., Odgren P.R. (2015). Studies of OC-STAMP in Osteoclast Fusion: A New Knockout Mouse Model, Rescue of Cell Fusion, and Transmembrane Topology. PLoS ONE.

[42] Yang M., Birnbaum M.J., MacKay C.A., Mason-Savas A., Thompson B., Odgren P.R. (2008). Osteoclast stimulatory transmembrane protein (OC-STAMP), a novel protein induced by RANKL that promotes osteoclast differentiation. J. Cell. Physiol..

[43] Kodama J., Kaito T. (2020). Osteoclast Multinucleation: Review of Current Literature. Int. J. Mol. Sci..

[44] Yagi M., Miyamoto T., Sawatani Y., Iwamoto K., Hosogane N., Fujita N., Morita K., Ninomiya K., Suzuki T., Miyamoto K. (2005). DC-STAMP is essential for cell-cell fusion in osteoclasts and foreign body giant cells. J. Exp. Med..

[45] Lee S.H., Rho J., Jeong D., Sul J.Y., Kim T., Kim N., Kang J.S., Miyamoto T., Suda T., Lee S.K. (2006). v-ATPase V0 subunit d2-deficient mice exhibit impaired osteoclast fusion and increased bone formation. Nat. Med..

[46] Maile L.A., DeMambro V.E., Wai C., Lotinun S., Aday A.W., Capps B.E., Beamer W.G., Rosen C.J., Clemmons D.R. (2011). An essential role for the association of CD47 to SHPS-1 in skeletal remodeling. J. Bone Miner Res..

[47] Chernomordik L.V., Kozlov M.M. (2008). Mechanics of membrane fusion. Nat. Struct. Mol. Biol..

[48] Irie A., Yamamoto K., Miki Y., Murakami M. (2017). Phosphatidylethanolamine dynamics are required for osteoclast fusion. Sci. Rep..

