# Supplementary material for: Surfactin Inhibits Osteoclast Differentiation by Negatively Regulating the Elk1-AP-1-NFATc1 Axis
Source: Biomedicines. 2026 Jan 11;14(1):155. doi: 10.3390/biomedicines14010155 (PMC12838702; doi:10.3390/biomedicines14010155)
Supplement: Supplementary file 1 [file biomedicines-14-00155-s001.zip › biomedicines-4009035-supplementary.pdf]

Supplemental Figure S1

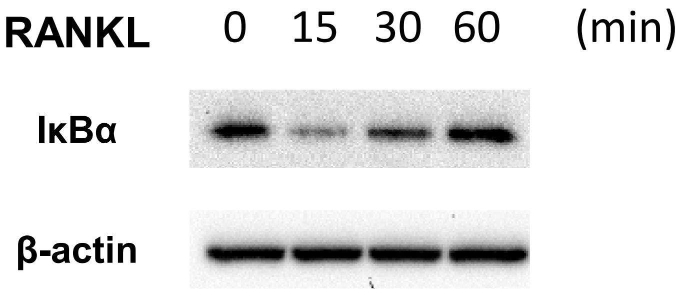

Figure S1. Time-dependent effects of RANKL on NF-κB activation.

Supplemental Figure S2

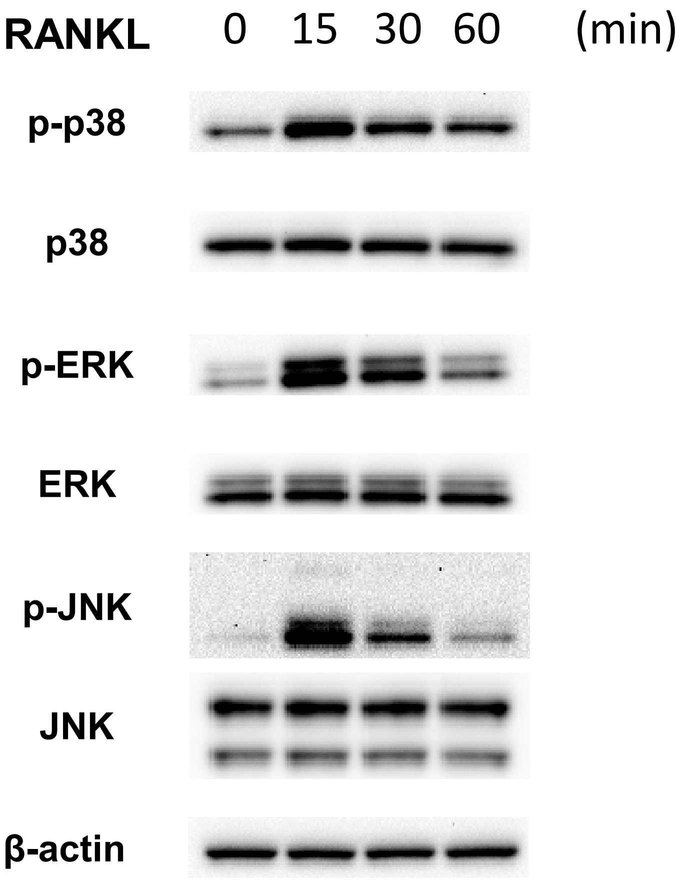

Figure S2. Time-dependent effects of RANKL on MAPK activation.

Supplemental Figure S3

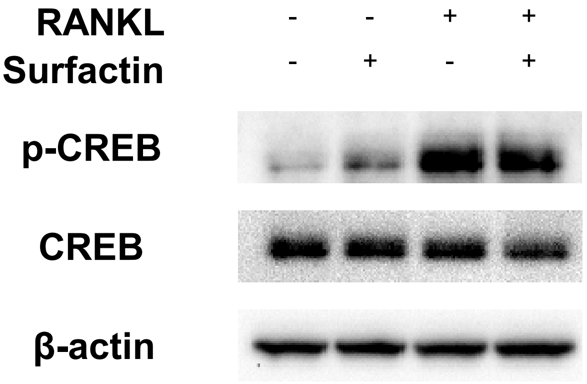

Figure S3. Effects of surfactin on CREB phosphorylation activated by RANKL.
